# Identification of *SERPINE1* as a Regulator of Glioblastoma Cell Dispersal with Transcriptome Profiling

**DOI:** 10.3390/cancers11111651

**Published:** 2019-10-25

**Authors:** Fidan Seker, Ahmet Cingoz, İlknur Sur-Erdem, Nazli Erguder, Alp Erkent, Fırat Uyulur, Myvizhi Esai Selvan, Zeynep Hülya Gümüş, Mehmet Gönen, Halil Bayraktar, Hiroaki Wakimoto, Tugba Bagci-Onder

**Affiliations:** 1 Brain Cancer Research and Therapy Laboratory, Koç University School of Medicine, Istanbul 34450, Turkey; fseker14@ku.edu.tr (F.S.); acingoz@ku.edu.tr (A.C.); nerguder14@ku.edu.tr (N.E.); merkent@ku.edu.tr (A.E.); 2 Department of Computational Biology, Koç University, Istanbul 34450, Turkey; firatuyulur@hotmail.com; 3 Department of Genetics and Genomic Sciences, Icahn School of Medicine at Mount Sinai, New York, NY 10029, USA; myvizhi.selvan@mssm.edu (M.E.S.); zeynep.gumus@gmail.com (Z.H.G.); 4 Icahn Institute for Data Science and Genomic Technology, New York, NY 10029, USA; 5 Department of Industrial Engineering, College of Engineering, Koç University, Istanbul 34450, Turkey; mehmetgonen@ku.edu.tr; 6 Department of Molecular Biology and Genetics, Istanbul Technical University, Istanbul 34467, Turkey; bayrakta.h@gmail.com; 7 Department of Neurosurgery, Massachusetts General Hospital, Harvard Medical School, Boston, MA 02114, USA; HWAKIMOTO@mgh.harvard.edu

**Keywords:** GBM, transcriptome analysis, dispersal

## Abstract

High mortality rates of glioblastoma (GBM) patients are partly attributed to the invasive behavior of tumor cells that exhibit extensive infiltration into adjacent brain tissue, leading to rapid, inevitable, and therapy-resistant recurrence. In this study, we analyzed transcriptome of motile (dispersive) and non-motile (core) GBM cells using an in vitro spheroid dispersal model and identified SERPINE1 as a modulator of GBM cell dispersal. Genetic or pharmacological inhibition of SERPINE1 reduced spheroid dispersal and cell adhesion by regulating cell-substrate adhesion. We examined TGFβ as a potential upstream regulator of *SERPINE1* expression. We also assessed the significance of SERPINE1 in GBM growth and invasion using TCGA glioma datasets and a patient-derived orthotopic GBM model. *SERPINE1* expression was associated with poor prognosis and mesenchymal GBM in patients. SERPINE1 knock-down in primary GBM cells suppressed tumor growth and invasiveness in the brain. Together, our results indicate that SERPINE1 is a key player in GBM dispersal and provide insights for future anti-invasive therapy design.

## 1. Introduction

Glioblastoma multiforme (GBM) is the most common and malignant primary brain tumor [1]. Despite advances in diagnosis and treatment regimens, life expectancy still remains at approximately 12–18 months [2]. High mortality rates can be partly attributed to the incomplete surgical removal of tumor as tumor borders are diffuse, and individual cells that have infiltrated into the healthy parenchyma are not easily detectable [3]. Tumor cells that show extensive dispersal out of the primary tumor core lead to rapid and inevitable recurrence [4], due to the treatment-resistant nature of these cells [5]. Therefore, understanding the mechanisms of GBM cell invasion and developing anti-invasive therapies are of utmost importance for successful eradication of GBMs and improving patient prognosis [5,6].

Epithelial to mesenchymal transition (EMT) has an important role in cancer progression, where it controls the transcriptional programs operating during the transition between tumor growth and metastasis. As opposed to most carcinomas, GBM tumors show local invasion and dispersal within the brain tissue instead of distant metastasis. However, invasive GBMs share common molecular features with metastatic cancers [7] and some essential regulators of EMT, such as TGFβ, strongly stimulates GBM invasion [8]. An important mechanism supporting EMT and cancer cell invasion is the changes in cell adhesion. Indeed, local detachment of tumor cells from the primary tumor and their interaction with the adjacent parenchymal tissues facilitate their distant movement [9]. Adhesive properties of cancer cells are significant determinants of their invasive potential; and many adhesion-related proteins have been proposed as potential targets to inhibit invasion [10].

Examining the molecular events that underlie the invasion process of GBM cells is vital to understand the aggressive nature of GBMs. To this end, several reports have addressed the molecular mechanisms of tumor cell movement [2,11,12,13,14,15,16]. However, a thorough characterization of gene expression dynamics in an in vitro system that mimics tumor dispersal has not been performed. In this study, we investigated the transcriptome of motile (dispersive) and non-motile (core) GBM cells in a spheroid dispersal model that recapitulated the dynamic features of dispersal and tumor invasion. We showed that, besides genes that operate in cell proliferation, several EMT program genes, including *SERPINE1*, were markedly upregulated in dispersive population. Using loss-of-function approach, we showed that SERPINE1 silencing reduces GBM cell dispersal and the interactions of GBM cells with the extracellular environment. Our results suggest that SERPINE1 is a key player in GBM dispersal providing insights into the future design of anti-invasive therapies.

## 2. Results

### 2.1. Transcriptome Profiling of Motile and Non-Motile GBM Cells Reveal Major Alterations in Cell Proliferation and Movement Pathways

To generate an in vitro model that better mimics the dynamics that operate between the tumor core and tumor rim, we formed tumor spheroids and assessed their outward migration ability, here termed dispersal. Accordingly, we first assessed the sphere forming ability (Supplementary Figure S1A) and dispersal capacity (Supplementary Figure S1B) of six different GBM cell lines. We observed that LN18, LN229, and T98G cells stayed as multicentric clumps in the hanging drops and they were unable to form spheroids. On the other hand, A172, U373, and U87MG cells could form single compact spheroids. In this study, we worked with U373 cells which exhibited the highest dispersal capacity and A172 cells that have a modest dispersal capacity. In order to collect adequate amount of high-quality RNA from the dispersive cells, we used U373 for transcriptome profiling and verified the hits for both cell lines. We also utilized a patient-derived primary cell line, GBM8, to verify our phenotypical findings.

**Figure 1:**
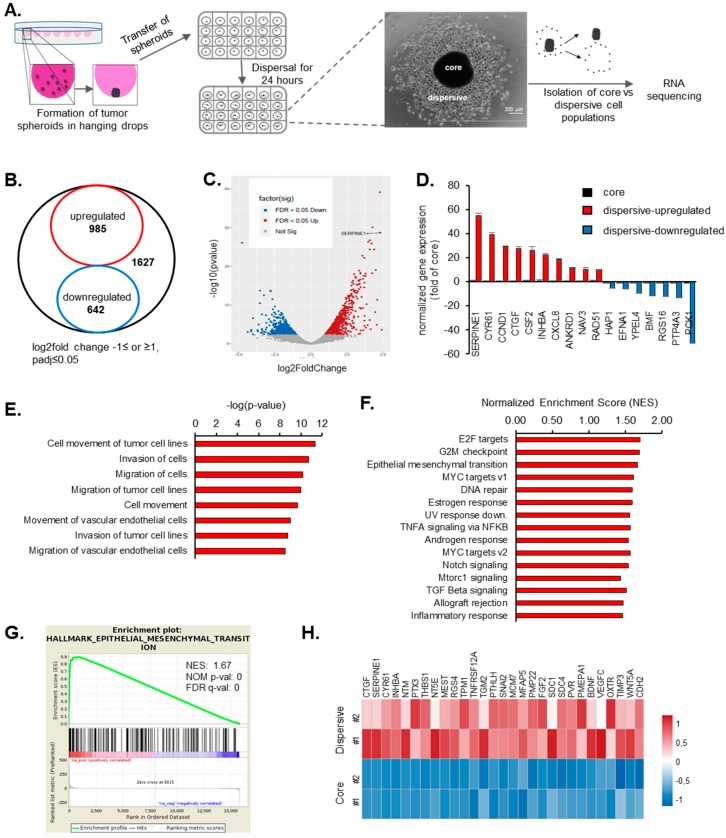
Transcriptome of motile and non-motile cells have major differences and *SERPINE1* is the top upregulated gene in dispersive cells. (**A**) hanging drops method was used to generate tumor-mimicking spheroids. After formation of tumor spheroids in hanging drops, spheres were transferred to 24-well plates and allowed to disperse for 24 h. Core and dispersive cells were collected separately for RNA sequencing. (**B**) total 1627 genes were differentially expressed between motile and non-motile cells (log2 fold change -1≤ or ≥1, *padj* ≤ 0.05); (**C**) volcano plot showing the upregulated (red) and downregulated (blue) genes in dispersive cells; (**D**) qRT-PCR validation of top differentially expressed genes in core and dispersive cells; (**E**) “Diseases and bio functions” from IPA core functional analysis of the differentially expressed genes related to “cell movement” in the dispersive cells (*z*-score of >|2|); (**F**) top 15 cancer hallmark gene sets enriched in dispersive cell transcriptome in GSEA analysis (*NOM p* ≤ 0.05); (**G**) enrichment plot for EMT gene set; (**H**) gene expression heat map of EMT genes in core and dispersive cells (biological duplicates were shown).

To assess the transcriptional differences between the core (non-motile) and dispersive (motile) cell populations, we manually isolated those cells that have dispersed and those have remained in the tumor cores after 24 h (Figure 1A). RNA-seq of core and dispersive cells pointed to major differences in transcriptome with 1627 differentially expressed genes (DEGs) (Figure 1B,C and Supplementary Figure S2). Of these DEGs, 985 were upregulated, and 642 were downregulated in dispersive cells. The differences in gene expression of the most significantly altered genes were validated with qRT-PCR in independently collected samples (Figure 1D). Functional analysis of RNA-seq results with Ingenuity Pathway Analysis (IPA) tool showed that “cell movement” was a majorly activated pathway as it was statistically significant in multiple disease and pathway sets (Figure 1E). Similarly, gene set enrichment analysis (GSEA) revealed that, in addition to several gene sets related with cell cycle such as “E2F targets”, “G2-M checkpoint”, and “Myc targets”, a movement related “EMT” gene set was significantly upregulated in dispersive cells (Figure 1F–H). Among the EMT related genes that were altered, *SERPINE1* was the most upregulated gene (Figure 1D). Other top upregulated genes linked with EMT were *CTGF* and *CYR61,* whose relations to GBM cell invasion were previously demonstrated [2], attesting to the strength of our approach for identifying mediators of dispersal. Indeed, downregulation of *CTGF* or *CYR61* reduced the dispersal ability of GBM cells in our spheroid model (Supplementary Figure S9) validating the findings of previous reports.

### 2.2. SERPINE1 Inhibition Reduces GBM Dispersal

**Figure 2:**
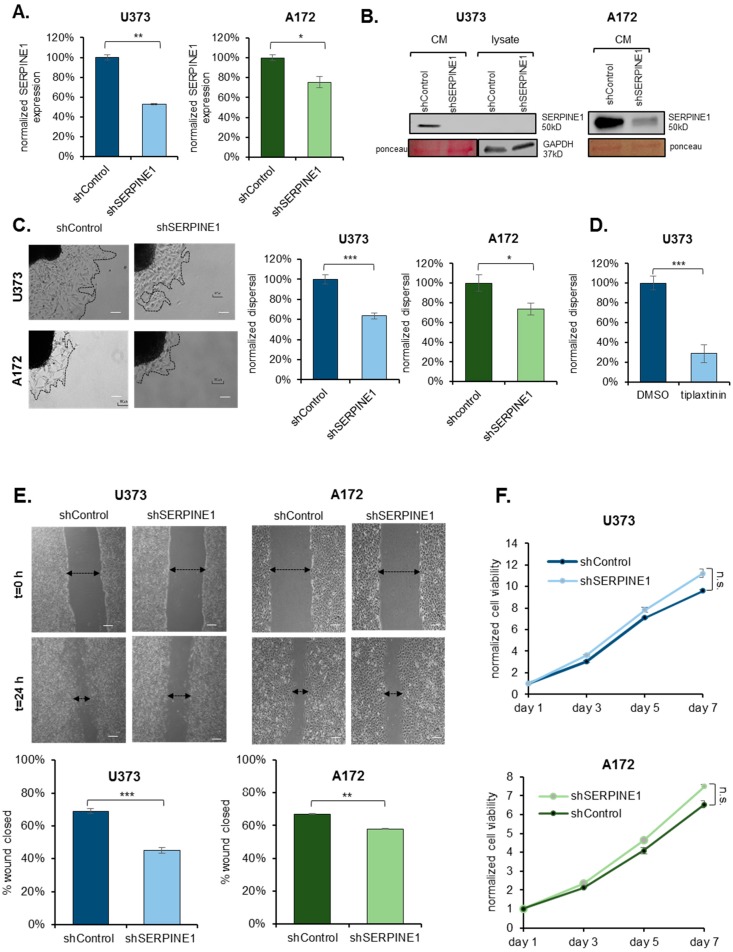
*SERPINE1* knock-down reduces GBM dispersal (**A**) qRT-PCR analysis of *SERPINE1* expression levels after shRNA knock-down; (**B**) SERPINE1 protein levels after shRNA knock-down; (**C**) dispersal assay that shows *SERPINE1* knock-down reduces dispersal of U373 and A172 spheroids significantly (*n* = 24 spheroids for each condition, scale bar: 200 µm); (**D**) dispersal assays that shows chemical inhibitor of SERPINE1, tiplaxtinin, reduces dispersal of U373 spheroids (*n* = 12 spheroids for each condition, scale bar: 200 µm); (**E**) wound healing analysis of the effect of *SERPINE1* knock-down (*n* = 35 areas for each condition, scale bar: 200 µm); (**F**) cell viability analysis of the effects of *SERPINE1* knock-down in U373 and A172 cell growth. (*, ** and *** denote *p* < 0.05, *p* < 0.01 and *p* < 0.001 respectively, two-tailed Student’s *t*-test).

Given the marked upregulation of *SERPINE1* in dispersive cells, we examined its function in GBM dispersal. To this end, we employed multiple GBM cell lines (U373 and A172), which both displayed *SERPINE1* upregulation in the dispersive cell population (Figure 1D, Supplementary Figure S3B), and have different endogenous SERPINE1 expression levels (Supplementary Figure S4A). These cells also display mesenchymal characteristics as shown by the expression of select epithelial and mesenchymal genes compared to an epithelial cancer cell line (Supplementary Figure S4B). Using multiple *SERPINE1* shRNAs, we were able to achieve significant *SERPINE1* silencing in both cell lines, as revealed by qRT-PCR and Western Blots (Figure 2A,B and Supplementary Figure S5A). Cells with *SERPINE1* knock-down showed significantly reduced dispersal (Figure 2C and Supplementary Figure S5B). This was not accompanied by changes in the overall mesenchymal state of cells as silencing of SERPINE1 did not markedly change the expression of selected mesenchymal genes, including *TWIST, SNAIL, N-CADHERIN,* and *SLUG*. However, there was a slight decrease in *WNT5A* expression upon SERPINE1 silencing (Supplementary Figure S6). In parallel, pharmacologic inhibition of SERPINE1 with a chemical inhibitor, Tiplaxtinin, led to a significant decrease in dispersal of U373 cells in accordance with the observed effects of genetic manipulation (Figure 2D) without affecting cell viability (Supplementary Figure S8A). These phenotypes were also observed in wound healing assay, where cells were first cultured to confluence and then induced to migrate by forming a scratch in the monolayer (Figure 2E). To test whether the reduced dispersal or migration is due to a decrease in cell proliferation, we analyzed the effect of *SERPINE1* knock-down on cell viability and observed comparable proliferative capacities of cells over seven days (Figure 2F). On the other hand, cells with reduced expression of cell cycle regulators, *CDC45*, and *MCM3* (Supplementary Figure S10B), which were also enriched in the dispersive cells as part of the “G2M checkpoint” and “E2F targets” gene set (Supplementary Figure S10A), showed reduced viability (Supplementary Figure S10C) and reduced dispersal (Supplementary Figure S10D). The changes in cell cycle of these cells were in line with the viability results, where more alterations in cell cycle were observed in shCDC45 and shMCM3 cells compared to shSERPINE1 or shControl cells (Supplementary Figure S10E). Together, these results suggest that the effects of *SERPINE1* knockdown on the dispersal of U373 or A172 cells were independent of cell viability changes.

### 2.3. SERPINE1 Knock-Down Reduces Cell Adhesion and Directional Persistence of GBM Cells

**Figure 3:**
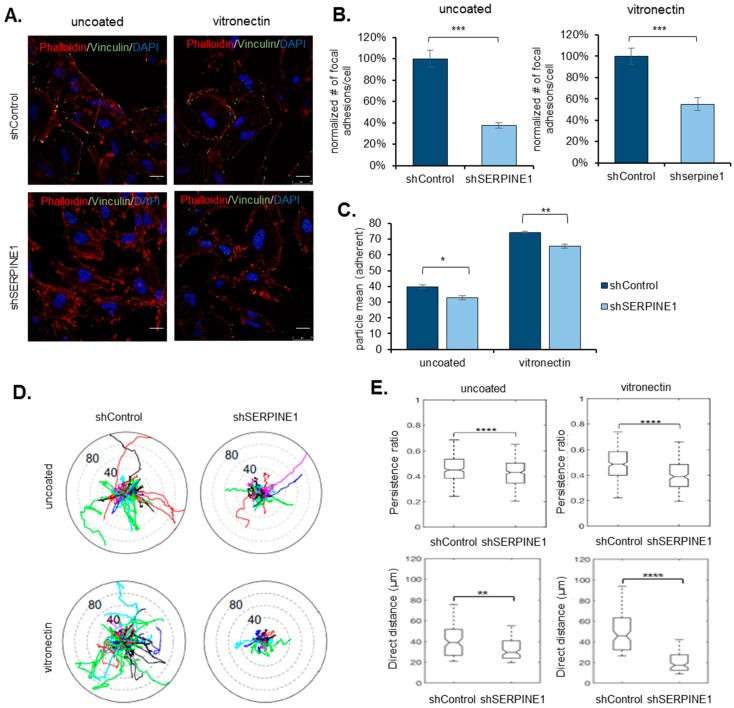
*SERPINE1* knock-down reduces cell adhesion and directional persistence of GBM cells (**A**) immunofluorescence staining for shControl and sh*SERPINE1* U373 cells (red: phalloidin, green: vinculin, blue: DAPI, scale bar: 200 µm) with/without vitronectin coating. (**B**) Analysis shows that the number of focal adhesions per cell is significantly reduced with *SERPINE1* knock-down (*n* = 20 cells analyzed for each condition). (**C**) Adhesion analysis for shControl and shSERPINE1 U373 cells (three wells/condition were analyzed, adherent particles were measured two hours after cell seeding); (**D**) polar plot obtained by tracking movement of individual shControl or shSERPINE1 cells with no coating or on vitronectin coating (>200 cells per condition tracked); (**E**) persistence ratio (top) or direct distance taken (bottom) for movements of shControl or shSERPINE1 cells with no coating or on vitronectin coating (>200 cells per condition tracked). (*, **, *** and **** denote *p* < 0.05, *p* < 0.01, *p* < 0.001 and *p* < 0.0001 respectively, two-tailed Student’s *t*-test).

Cell migration and dispersal are governed by the dynamic changes that occur at the contact points of cells with their extracellular environment, called focal adhesions. Indeed, motile cells display constant turnover of focal adhesions at their leading and trailing edges [9]. To investigate the mechanism by which SERPINE1 regulates dispersal, we examined focal adhesions using immunofluorescent staining for Vinculin, a known marker of focal adhesions. Accordingly, there was a remarkable reduction in the number of focal adhesions in *SERPINE1* knocked-down cells compared to controls (Figure 3A,B). This was coupled with a marked difference in the overall adhesion ability of cells, where the cells with *SERPINE1* knock-down were less adherent (Figure 3C). When the cells were subjected to vitronectin, an extracellular matrix protein and a co-factor for SERPINE1 [17], the reduction in the number of focal adhesions and cell adhesiveness were still evident (Figure 3A–C), noting that the number of focal adhesions per cell and overall adhesive nature was more prominent on vitronectin coating.

When the effects of SERPINE1 on dispersal were tested on Vitronectin-coating, Vitronectin alone increased dispersal (Supplementary Figure S11B) in U373 and A172 cells. On Vitronectin, *SERPINE1* knock-down decreased dispersal of U373 spheroids (Supplementary Figure S11D), but not of A172 spheroids (Supplementary Figure S11C). The basal expression of *SERPINE1* was not affected by vitronectin coating (Supplementary Figure S11A). To further dissect the effects of SERPINE1 on GBM cell motility, we tracked the movement of individual control and *SERPINE1* knock-down cells (Supplementary Videos S1–S4). Consistent with our previous findings described above, *SERPINE1* knock-down limited the movement of individual cells (Figure 3D) and markedly reduced the persistence and distance of cell movement (Figure 3E). These effects were also pronounced on Vitronectin-coating, suggesting that SERPINE1 facilitates cell migration and dispersal by regulating cell adhesion to the extracellular environment.

### 2.4. TGFβ Is an Upstream Regulator of SERPINE1

**Figure 4:**
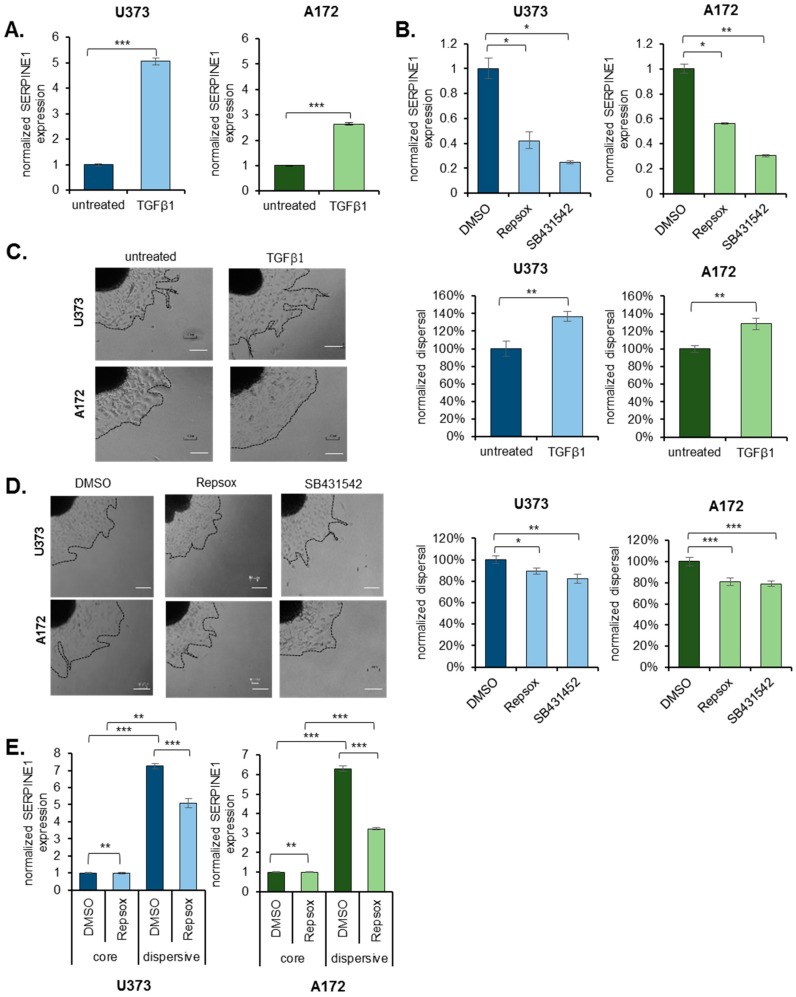
TGFβ is an upstream regulator of *SERPINE1***.** (**A**) qRT-PCR analysis of *SERPINE1* expression upon TGFβ treatment in U373 and A172 cells. (**B**) qRT-PCR analysis of *SERPINE1* expression upon TGFβ inhibitor (Repsox and SB431542) treatment in U373 and A172 cells; (**C**) dispersal assay that shows TGFβ induces dispersal of U373 and A172 (*n* = 12 spheroids for each condition, scale bar: 300 µm). (**D**) Dispersal assay that shows Repsox or SB431542 reduce dispersal of U373 and A172 spheroids (*n* = 24 spheroids for each condition, scale bar: 300 µm); (**E**) *SERPINE1* upregulation in dispersive cells in the presence of TGFβ inhibitor Repsox for U373 and A172 cells. (*, ** and *** denote *p* < 0.05, *p* < 0.01 and *p* < 0.001 respectively, two-tailed Student’s *t*-test).

Given the remarkable induction of *SERPINE1* during dispersal, we wished to examine the possible upstream regulators of *SERPINE1* expression. Based on the GSEA analysis, TGFβ signaling was activated in the dispersive population (Figure 1F, Supplementary Figure S12A,B). Since TGFβ is also a known regulator of EMT, we addressed whether it would change *SERPINE1* expression and ultimately cell dispersal. To this end, treatment of U373 or A172 cells with TGFβ caused a significant upregulation of *SERPINE1* expression in both cell lines (Figure 4A). On the contrary, inhibition of TGFβ signaling with two independent chemical inhibitors, Repsox or SB431542, decreased *SERPINE1* expression (Figure 4B) in these cells. In parallel with the changes in *SERPINE1* expression levels, dispersal of the spheroids was increased with TGFβ (Figure 4C) and decreased with TGFβ inhibitors (Figure 4D). To test whether the dynamic induction of *SERPINE1* is dependent on TGFβ signaling, we added TGFβ inhibitors on spheroids and assessed *SERPINE1* expression between core and dispersive cells. Accordingly, upregulation of *SERPINE1* in dispersive population was partly inhibited by TGFβ inhibitors (Figure 4E, Supplementary Figure S12C), demonstrating a regulatory role of TGFβ signaling in *SERPINE1* induction. 

### 2.5. SERPINE1 Expression Is Associated with Poor Patient Survival and Its Silencing in A Clinically-Relevant Model Reduces Dispersal

**Figure 5:**
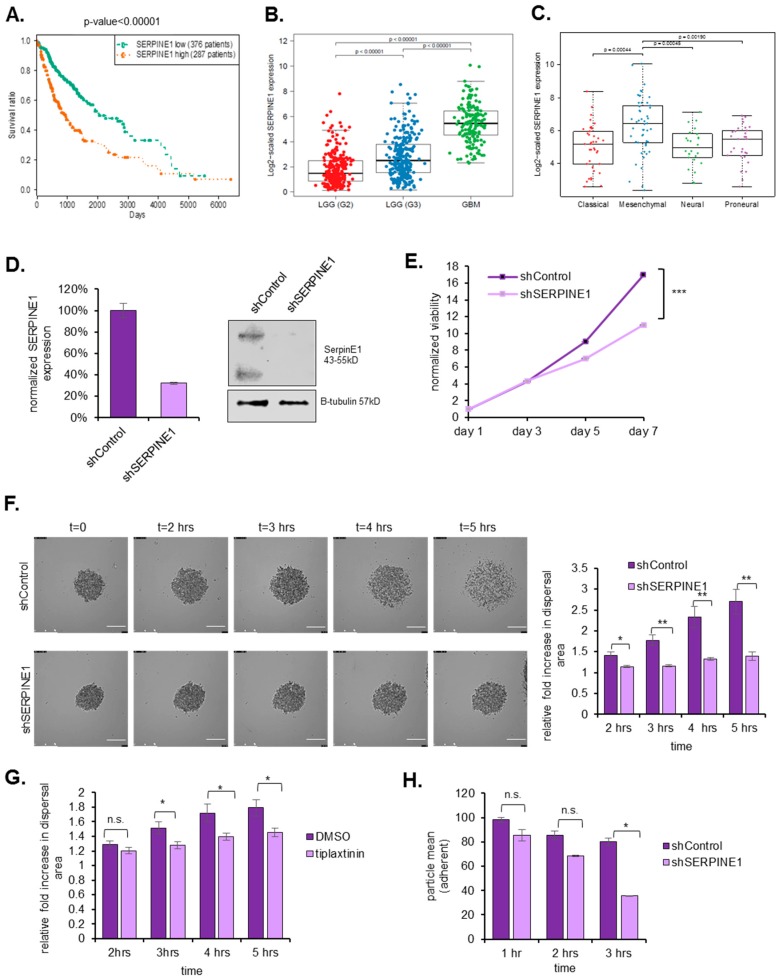
*SERPINE1* expression is correlated with poor prognosis and its silencing in a clinically-relevant model reduces cell viability and dispersal. (**A**) TCGA survival data plotted for high/low *SERPINE1* expressing glioma patients. (**B**) *SERPINE1* expression correlation with LGG samples and GBM samples; (**C**) *SERPINE1* expression correlation with GBM subtypes; (**D**) *SERPINE*1 mRNA and protein levels after shRNA knock-down in GBM8 cells; (**E**) cell viability assays that show *SERPINE1* knockdown slows down GBM8 cell proliferation; (**F**) live cell imaging and analysis of shControl and shSERPINE1 spheroids. *SERPINE1* knockdown reduces dispersal of GBM8 spheroids (video for five hours of dispersal, images taken every 5 min, scale bar: 200 µm, *n* = 10 spheroids for each condition); (**G**) live cell imaging analysis of DMSO- or Tiplaxtinin-treated GBM8 spheroids. (video for five hours of dispersal, images taken in every 60 min, magnification is 10×, *n* = 12 spheroids for each condition); (**H**) *SERPINE1* knock-down reduces the number of attached cells in different time points (three wells analyzed for each condition and each time point). (*, ** and *** denote *p* < 0.05, *p* < 0.01 and *p* < 0.001 respectively, two-tailed Student’s *t*-test).

To examine the clinical relevance of SERPINE1, we examined the relation of *SERPINE1* expression with patient survival in the TCGA datasets. Accordingly, in a total of 663 patient samples composed of low-grade glioma and GBM, Kaplan–Meier survival curves of the “*SERPINE1* high” and “*SERPINE1* low” groups revealed inverse correlation of *SERPINE1* with patient survival *(p* = 0.00014) (Figure 5A). In addition, *SERPINE1* expression correlated with increasing glioma grade (Figure 5B). Moreover, *SERPINE1* expression was mostly enriched in the mesenchymal subtype GBM (Figure 5C), which corresponds to poor survival, invasiveness, and therapy resistance in GBM [18].

To further examine the effects of SERPINE1 in a clinically-relevant model, we chose a patient-derived primary cell line, GBM8, which grows as neurospheres [19]. Following shRNA mediated knock-down (Figure 5D), we observed that *SERPINE1* silencing had a growth-slowing effect on GBM8 cells (Figure 5E). Using live-cell imaging to track motility of cells dispersing out of tumor spheres, we observed that *SERPINE1* knock-down reduced dispersal significantly in these cells in a short time window of five hours due to the highly invasive nature of this primary cell line (Supplementary Videos S5 and S6 and Figure 5F). Concomitantly, chemical inhibition of SERPINE1 with Tiplaxtinin reduced dispersal markedly in GBM8 cells (Figure 5G) and two other primary cell lines GBM4 and MGG119 (Supplementary Figure S7). Consistent with our previous observations, GBM8 cells with *SERPINE1* knock-down were less adherent than control cells (Figure 5H). Thus, the effects of SERPINE1 on dispersal was validated in a clinically relevant model.

### 2.6. SERPINE1 Knock-Down Reduces Tumor Progression In Vivo

**Figure 6:**
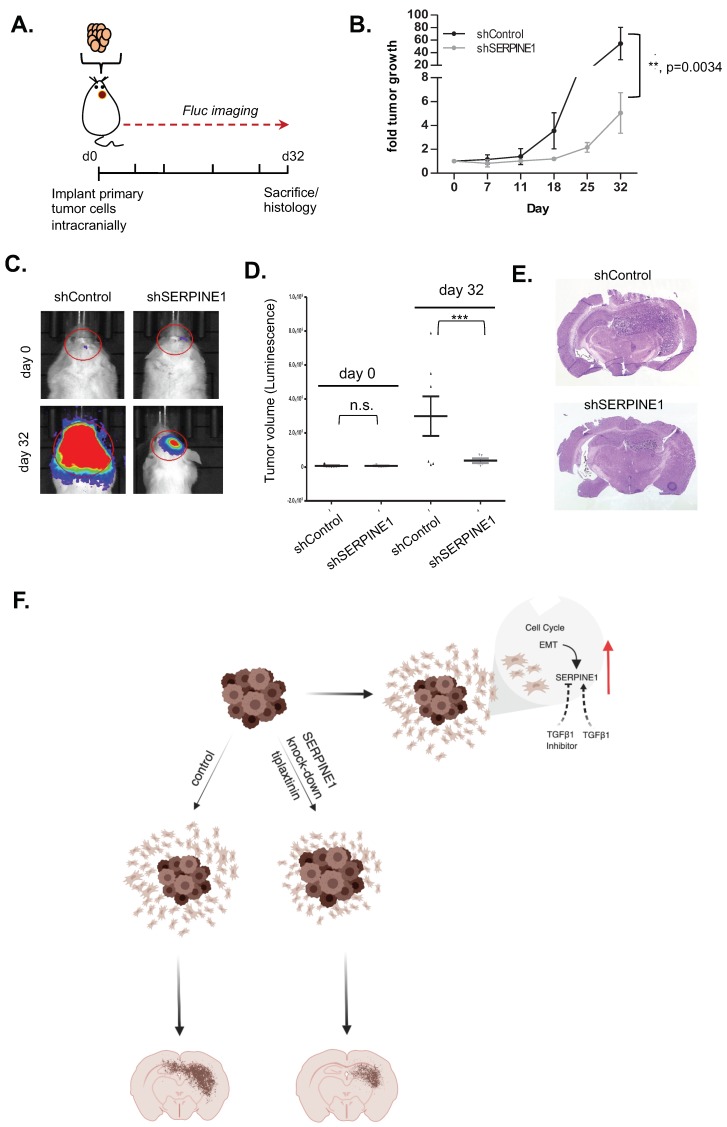
*SERPINE1* knock-down reduces tumor progression in vivo. (**A**) strategy of the in vivo experiment; (**B**) graph showing tumor growth as measured by bioluminescence radiance for 32 days after tumor cell injection. Data were normalized to day 0 signal of each group (*n* = 7 mice for shControl, *n* = 5 mice for shSERPINE1); (**C**) representative bioluminescence images of tumors from day 0 and 32 displaying normalized bioluminescent efficiencies acquired (blue to red indicates lower to higher radiance as photons/s/cm^2^/steradian); (**D**) plot depicting individual tumor volumes on day 0 and day 32; (**E**) representative H&E images of shControl and shSERPINE1 tumors (magnification is 13.5×); (**F**) model describing dynamic regulation of *SERPINE1* and mechanism of *SERPINE1* knock-down acting on dispersal. Image created with BioRender. (** and *** denote *p* < 0.05, *p* < 0.001 respectively, ANOVA).

To test the effect of *SERPINE1* knock-down on tumor growth, we used an orthotopic xenograft model of GBM8 cells transduced with shControl or shSERPINE1. To noninvasively monitor tumor growth, the cells were also transduced with a vector encoding firefly luciferase (Fluc) and mCherry (Figure 6A). Repeated bioluminescence imaging measurements revealed that the rate of growth of shSERPINE1 tumors was significantly lower than that of shControl tumors (Figure 6B–D). End-point histological examination of brain tumor sections showed that the overall sizes of shControl tumors were markedly larger than shSERPINE1 tumors and that individual shControl tumor cells invaded into distant sites in the brain parenchyma. In contrast, shSERPINE1 tumors remained small and appeared to have less distal invasion (Figure 6E). Taken together, these findings showed that *SERPINE1* silencing attenuated GBM growth and invasion in the brain in a clinically relevant in vivo model.

## 3. Discussion

High mortality rates of GBM patients are partly attributed to the invasive behavior of tumor cells, which show extensive infiltration into adjacent brain tissue leading to rapid and almost inevitable recurrence. Given the additional chemo- and radio-resistant characteristics of these invasive cells “left behind” after surgical resection, conventional therapies remain ineffective. Therefore, understanding the mechanisms of GBM cell invasiveness is of utmost priority to develop successful therapeutic approaches. In this study, we analyzed the dynamic changes in transcriptome of motile (dispersive) and non-motile (core) GBM cells and identified *SERPINE1* as a dramatically induced gene in the dispersive cell populations. We showed that genetic or pharmacological inhibition of SERPINE1 led to reduction of dispersal, attributing a functional role for SERPINE1 in dispersal. Furthermore, we demonstrated that SERPINE1 regulates cell-substrate adhesion and directional movement of GBM cells, and that its expression is regulated by TGFβ signaling (model in Figure 6F). Together, our results suggest that SERPINE1 is a key player in GBM dispersal providing insight into the future design of anti-invasive therapies.

The approach we employed in this study was transcriptome profiling of dispersive cells in a spheroid model, which mimics the three-dimensional tumor environment and outward cell migration [20]. With this approach, we provided a motility signature of GBM cells, and demonstrated that cell proliferation and migration programs were coupled in dispersal. Our approach was in accordance with previous reports [2]. Comparative studies that utilized laser capture microdissection followed by microarray analysis identified signature differences in tumor cores vs. infiltrating cells [11,12]. In addition to comparative studies, functional studies with genetic or proteomic approaches were conducted to discover regulators of tumor cell movement. Accordingly, expression screens [13,14], RNAi-based loss-of-function screens [15,21], and proteomic screens [22] have already identified several novel regulators of tumor cell migration. Consistent with these studies, our study identified a larger number of differentially expressed genes, most of which were upregulated during dispersal, revealing dynamic and adaptable transcriptome of moving cells. Our approach does not directly test the causality or functionality of the altered genes; however, it provides a groundwork and several candidate networks to examine in detail. Indeed, our study identified several markedly upregulated genes, some of which were previously shown, supporting the validity and strength of our approach. Notably, *CTGF* and *CYR61* genes were defined as part of “migratory signature” [2], with expression changes and functionality validated in our model. In addition, we identified many altered cell cycle related gene sets in dispersive cells. Most cell division and proliferation related genes were upregulated, suggesting that cells that disperse out of spheres can also alter their gene expression in favor of growth. This finding is in contrast with the model of dichotomy between migration and proliferation [23], which suggests that proliferation and migration are mutually exclusive. Dissecting the interplay between dispersal and proliferation with single-cell based assays will be crucial to address these questions that remain to be resolved.

We observed that the EMT gene set was significantly upregulated in dispersive cells. While EMT and distant metastasis is not readily observed in GBMs, invasive GBMs share common molecular features with metastatic cancers [7]. Indeed, GBMs that undergo mesenchymal transition are associated with a more aggressive and treatment-resistant phenotype [18]. Within EMT genes that were upregulated in dispersive cells, *SERPINE1* had the highest levels reaching up to 36-fold of core cells at 24 h. Furthermore, the induction of *SERPINE1* expression was persistent (Supplementary Figure S3A) suggesting a critical role for it in GBM cells dispersal. *SERPINE1* is a member of the serine proteinase inhibitor (serpin) superfamily and also known as a plasminogen activator inhibitor (PAI-1) [24]. Being a regulator of plasminogen activator system, *SERPINE1* has a central role in ECM degradation and remodeling [24] as well as cell migration in different physiological conditions [25]. Indeed, high levels of *SERPINE1/PAI*-1 have been correlated with poor prognosis in several cancer types [26,27]. Recent studies showed that *SERPINE1* expression is correlated with glioma grade [28] and that SERPINE1 is found in the unique proteomic signature of mesenchymal subtype of GBMs [29]. These reports are in line with our demonstration that high levels of *SERPINE1* expression strongly correlate with poor survival in glioma patients in the TCGA cohort. In addition, we show that *SERPINE1* is highly enriched in mesenchymal subtype of GBM, which corresponds to poor survival and a resistant phenotype in GBM [18]. Overall, SERPINE1 is a strong prognostic indicator for GBM and might play a critical role in its progression through mechanisms that are largely unresolved.

While the role of SERPINE1 in cell migration has been explored in non-malignant contexts, such as epithelial cells [30,31,32,33,34,35], its specific role in GBM cells has been elusive. There have been few reports indirectly linking SERPINE1 expression to GBM progression. For example, a recent study showed that *SERPINE1* is a target of a microRNA (miR-1275) that regulates proliferation and invasion of glioma cells [36]. Another report suggested that GBM cell *SERPINE1* expression is controlled by GDF-15, a cytokine in the TGFβ superfamily [37]. To our knowledge, our study provides the first functional demonstration of a direct role of SERPINE1 in GBM cell motility as well as a pro-tumorigenic role in in vivo GBM models. Indeed, silencing of SERPINE1 or its pharmacological inhibition reduced the migration and dispersal of GBM cells in vitro, as well as tumor growth in a primary GBM model in vivo. Therefore, SERPINE1 is a potential target for anti-GBM therapies in the future.

Regulation of cell adhesion to extracellular matrix is an important component of tumor cell invasion, where the cells generate or breakdown receptor-mediated focal adhesion points in the direction of cell movement [38]. We demonstrate that SERPINE1 is a critical regulator of the adhesion process, as the number of focal adhesions and directionality of cell movement on vitronectin, a known interactor of SERPINE1 [39], was greatly affected by SERPINE1 silencing in our models. This is in accordance with previous findings that showed that SERPINE1 regulated adhesive behavior of smooth muscle cells [40], or fibrosarcoma cells [41].

How *SERPINE1* gene expression is regulated is an interesting question, given its marked elevation during GBM dispersal. Assessing upstream molecular events might be crucial to find novel anti-invasive approaches. To this end, our study demonstrated that TGFβ signaling is a critical regulator of *SERPINE1* expression in GBM cells. Indeed, treatment with TGFβ or TGFβ inhibitors markedly regulated *SERPINE1* expression and dispersal. This is in consistence with previous findings on regulation of *SERPINE1* expression [42,43], and is also supported by our IPA analysis that identified Smad2 and Smad3 as potential upstream regulators of *SERPINE1* expression in dispersive cells (Supplementary Figure S12A).

Despite the accumulating knowledge on the biology of invasive cells in GBMs, there is no therapy directed against these populations [3]. Even worse, current therapeutic strategies such as fractionated radiation further increase invasiveness of the cells [44]. As anti-invasive strategies, ephrin receptors, Rho GTPases and casein kinase 2 were considered as druggable targets [45]. In addition, inhibiting Matrix Metalloproteinases (MMPs) can be a good approach; however, application of MMP inhibitors in clinical trials did not improve patient survival when combined with temozolomide [46]. Other trials targeting integrins failed to show significant survival benefit in Phase III [47]. Currently, various TGFβ inhibitors, including Galunisertib in combination with standard therapy are being tested in glioma patients [45]. Our identification of SERPINE1 as a mediator of GBM progression provides another member to the growing list of therapy targets. Given its well-established clinical relevance, and, in light of our findings, inhibition of SERPINE1 may be a promising anti-invasive strategy for GBM.

## 4. Materials and Methods

### 4.1. Cell Culture and Reagents

A172 and U373, LN18, LN229, T98G and U87MG GBM cell lines and human embryonic kidney 293T cells were obtained from American Tissue Type Culture Collection (Manassas, VA, USA) and cultured in DMEM medium (Gibco, Gaithersburg, MD, USA) with 10% fetal bovine serum and 1% Penicillin-Streptomycin (Gibco, Gaithersburg, MD, USA). GBM8, GBM4 and MGG119 cells [19,34] were cultured in neurobasal medium (Gibco, Gaithersburg, MD, USA) supplemented with 3 mM L-Glutamine (Mediatech/Sigma-Aldrich, Woburn, MA, USA), B27 (Invitrogen/Gibco, Norcross, GA, USA), 2 μg/mL heparin (StemCell Technologies/Fisher Scientific, Kent, WA, USA), 20 ng/mL human EGF (R&D Systems, Minneapolis, MN, USA), and 20 ng/mL human FGF-2 (PeproTech Hamburg, Germany) (EF media). All cells were grown in 37 °C, 5% CO_2_ in a humidified incubator. Vitronectin (Gibco, Gaithersburg, MD, USA), Collagen (Gibco, Gaithersburg, MD, USA), recombinant human TGFβ1 (Peprotech 100-21, Hamburg, Germany), Tiplaxtinin (Selleckchem PAI-039, Houston, TX, USA), Repsox (Tocris, Ellisville, MO, USA), and SB431542 (Stemcell Technologies, Kent, WA, USA) were used for dispersal experiments. D-luciferin was used for in vivo imaging (Biotium, Fremont, CA, USA).

### 4.2. Generation of Tumor Cell Spheroids

For generating A172 and U373 spheroids, cell suspensions of 20,000 cells/20 µL drops were generated in DMEM medium with 10% FBS; and drops were placed on the cover of a 10 cm culture plate. Covers were flipped to allow for hanging drop formation, which were incubated at 37 °C incubator for 3 days in order to generate spheres. Using 200 µL pipette tips, spheroids were manually transferred to 24-well plates for dispersal experiments. Spheroids were allowed to disperse in DMEM with 10% FBS media. Shape coefficients of spheres were determined using ImageJ software (NIH Image, Bethesda, MD, USA). For GBM8, GBM4, and MGG119 neurospheres, EF media was used, and spheres were naturally generated in suspension.

### 4.3. Dispersal Assays

Tumor spheres were allowed to settle and attach to 24-well plates in culture medium. Cells were allowed to disperse out of sphere for 24 h if otherwise stated. For dispersal assays of A172 and U373 spheroids, DMEM medium supplemented with FBS was used. For dispersal assays of GBM8, GBM4 and MGG119 spheroids, EF medium was used. For assays with *SERPINE1* pharmacological inhibition, U373 spheres and primary cell line spheres (GBM8, GBM4, MGG119) were treated with 300 µM or 25 µM tiplaxtinin for 24 h, respectively. For assays with vitronectin coating, vitronectin was diluted 1:1000 with PBS, surface was coated at 37 °C for 2 h. For assays conducted with collagen coating, collagen was diluted 1:60 with 0.02 N acetic acid, and surface was coated at 37 °C for an hour. For assays testing TGFβ signaling, spheres were treated with TGFβ (50 ng/mL), Repsox (1 µM for U373, 5 µM for A172) or SB431542 (2.5 µM) for 24 h after attachment. Images were taken using Nikon Eclipse TS100 Inverted Fluorescence Microscope (Nikon Instruments Inc., Melville, NY, USA).

### 4.4. Dispersal Area Analysis

Dispersal area analysis was performed using paint.net software (San Francisco, CA, USA). Images (an image corresponding to each sphere, 12 or 24 spheres per condition) were analyzed using a Lasso tool. Total area of dispersal and remaining spheroid were measured, and overall dispersal was determined using the following equation:

$$\mathrm{dispersal}\;\mathrm{area}=\frac{\left[\mathrm{total}\;\mathrm{area}\left(24\;\mathrm{hours}\right)-\mathrm{sphere}\;\mathrm{area}\left(24\;\mathrm{hours}\right)\right]}{\left[\mathrm{sphere}\;\mathrm{area}\left(\mathrm{time}\;0\right)\right]}\;.$$

### 4.5. Wound Healing Assays

For wound healing experiments, 400,000 cells/well were seeded on 6-well plates. Cells were scratched using a 200 µL tip, washed with PBS and media was refreshed. Images were taken using Nikon Eclipse TS100 Inverted Fluorescence Microscope (Nikon Instruments Inc., Melville, NY, USA). Multiple images were collected from the wound 24 h after stratching. Distance of the cells from each side of the wound were analyzed using ImageJ (*n* = 35 areas were analyzed for each condition).

### 4.6. RNA Sequencing (RNA-seq) and Transcriptome Profiling of Core and Migratory Cells

For the experiments, 360 spheroids for each condition were allowed to disperse for 24 h. Core and dispersive populations were collected by manual dissection. Separately collected dispersive cells and cores were pelleted by centrifugation and RNA was isolated using Macharey–Nigel RNA kit (Düren, Germany) following manufacturer’s instructions. Library preparation, sequencing, and raw data processing were performed at the Epigenomics Core at Weill Cornell Medical School, Genomics Core Facility (New York, NY, USA). Briefly, RNA-seq libraries were prepared using established Illumina methods (Part #RS-122-2001), using HiSeq2500 (Illumina, San Diego, CA, USA). Single end 50 bp reads were generated with 2 biological replicates for each condition. Primary processing of sequencing images was done using Real-Time Analysis software (RTA) (Illumina, San Diego, CA, USA). CASAVA 1.8.2 software (Illumina, San Diego, CA, USA) was then used to demultiplex samples, generate raw reads and respective quality scores, as well as to perform image capture, base calling, and demultiplexing.

For RNA sequencing analysis, single-end reads were aligned to human genome GRCh38 using an HISAT2 [48] aligner using prebuilt indexes that were downloaded from the official website of HISAT2. The resulting sam format files were converted to bam and sorted using SAMtools [35]. The aligned reads were counted using FeatureCounts [49]. Differentially expressed genes were identified based on negative binomial distribution using DESeq2 (v.1.18.1) [50]. The RNA-seq data have been deposited in NCBI’s Gene Expression Omnibus (GEO), with accession number GSE130857. Enrichment of gene sets and functions were analyzed using Ingenuity Pathway Analysis (IPA) [51] and Gene Set Enrichment Analysis (GSEA) [52] software (GSEA V4.0.2, Cambridge, MA, USA).

### 4.7. qRT-PCR Experiments

qRT-PCR experiments were conducted using SYBR Green and LightCycler480 (Roche, Indianapolis, IN, USA) as described [53]. Primer sequences used in qRT-PCR experiments are given in Supplementary Table S1.

### 4.8. Cloning and Packaging of Silencing Vectors

shRNA sequences were designed using an RNAiCodex program [54]. shRNA sequences targeting related genes are given in Supplementary Table S2. Oligos were PCR-amplified by using following primers having compatible restriction ends with backbone vector, pSMP. Forward: 5′-GATGGCTGCTCGAGAAGGTATATTGCTGTTGACAGTGAGCG-3′, Reverse: 5′-CCCTTGAACCTCCTCGTTCGACC-3′. PCR products were cloned into an pSMP retro-viral backbone as described [55]. All vectors were verified by sequencing and packaged into retroviral particles as described [55,56].

### 4.9. Western Blotting

Conditioned medium (CM) or cell lysates derived from A172, U373, or GBM8 cells were used to examine SERPINE1 protein levels. For CM collection, media on cells growing in culture were refreshed with serum-free DMEM. After 48 h of incubation, CM and cell lysates were obtained. GBM8 cells were seeded with EF media and cultured for 48 h before CM and lysate collection. CM was added to a 10 kDa ultrafiltration tube (50 mL, Millipore, Dachstein, France) and centrifuged at 3500× *g* in 4 °C, for 30 min for enrichment. Protein extraction and Western blotting was performed as described [57]. The following primary antibodies were used: SERPINE1 (sc-5297 Santa Cruz Biotechnology, Santa Cruz, CA, USA), GAPDH (ab9485 Abcam, Cambridge, MA, USA), Beta-tubulin (ab6046 Abcam). Panceu staining of the *PVDF* membranes were also used to check for equal loading of CM. Secondary antibodies against corresponding antibodies were horseradish peroxidase coupled (1:10,000, Cell Signaling Technologies, Danvers, MA, USA). Blots were incubated with Clarity^TM^ Western ECL Substrate (Biorad, Philadelphia, PA, USA) and visualized using an Odyssey Scanner (LiCor Biosciences, Lincoln, NE, USA). Detailed information about western blot can be found at supplementary Figure S13.

### 4.10. Cell Viability Experiments

Cell viability was measured with ATP based Cell Titer-Glo (CTG) Luminescent Cell Viability Assay (Promega, San Luis Obispo, CA, USA) according to the manufacturer’s instructions using a plate reader (BioTek’s Synergy H1, Winooski, VT, USA). In addition, 1000 cells/well were seeded to 96-well plates (Corning Costar, clear bottom black side, Harrodsburg, KY, USA) as triplicates for each condition and cell growth was determined by repeated measurement of cell viability on days 3, 5 and 7 after seeding.

### 4.11. Immunofluorescence Staining

Cells were fixed with 3% PFA for 5 min. Fixed cells were washed with PBS and permeabilized with 0.1× Triton. After washing and blocking, coverslips were incubated with primary antibodies at 4 °C overnight and with secondary antibodies at room temperature for 1 h. Mounting was performed with VectaShield (Vector Laboratories, Burlingame, CA, USA) with DAPI. Antibodies used include: anti-vinculin antibody (ab73412, Abcam), and AlexaFluor488 rhodamine-phalloidin (Thermo R415). Images were taken using Leica DMI8 SP8 CS/DLS microscope (Leica Microsystems, Wetzlar, Germany) at 63× magnification. At least 20 cells were analyzed for each condition.

### 4.12. Adhesion Experiments

In addition, 100,000 cells/well were seeded on 24-well plates and allowed to adhere. Unattached cells were washed off with PBS and attached cells were fixed with ice-cold methanol at different time points (at 10 min intervals up to 3 h) after seeding. Attached cells were stained with crystal violet (Sigma) for 1 h, washed and left to dry. Plates were scanned and particle mean for each well were analyzed using Adobe Photoshop (Berkeley, CA, USA). Triplicates were used for each condition. To ensure the seeding of equal number of cells/well from each group, starting cell suspensions were subjected to viability assays and consistency in the cell number was verified.

### 4.13. Single-Cell Tracking and Persistence Analysis

The trajectories of cells on uncoated and vitronectin-coated surfaces were determined by using a custom script written in MATLAB (R2017b, Mathworks, Natick, MA, USA). Single-cell tracking code to determine time-dependent positions of cells was partially adapted from previous studies [58,59]. Briefly, point defects were removed by using a Gaussian filter with a lower bound of 3 pixels. A threshold filter was applied to determine the location of each cell. Centroid position of segmented cells was later determined by comparing intensity values in the neighboring pixels. Mean square displacement of cell position in consecutive frames was computed to associate each cell. Trajectories of cells were displayed on a polar plot. Persistence ratios of cells were analyzed by computing the ratio of direct distance to total displacement. If the ratio approaches to 1, cells tend to move linearly. Low persistence ratios imply a random migration. Direct displacement was measured by an interval of 8 frames while cumulative displacement was computed by an interval of 2 frames to avoid the overestimation of persistence due to the movement of cell centroid positions.

### 4.14. Patient Survival Analysis

Gene expression profiles of “glioblastoma multiforme” (GBM) and “brain lower grade glioma” (LGG) tumors were preprocessed by the unified RNA-Seq pipeline of The Cancer Genome Atlas (TCGA) consortium. For both cancer types, HTSeq-FPKM files of all primary tumors from the most recent data freeze (i.e., Data Release 14–December 18, 2018) were downloaded, leading to 703 files total. Clinical annotation files of cancer patients were used to extract their survival characteristics (i.e., days to last follow-up for alive patients and days to death for deceased patients). Clinical Supplement files of all patients from the most recent data freeze were downloaded, leading to 1114 files in total. To perform survival analysis using gene expression profiles, a total of 663 patients with survival information and gene expression profile available were included. The gene expression profiles of primary tumors were first log_2_-transformed and then *z*-normalized within each cohort before further analysis. For analyses, 663 samples were grouped into two categories (i.e., low and high) based on comparing each sample’s gene expression value compared to the mean expression value of that particular gene. Kaplan–Meier analysis was used to compare the survival of these two groups and the log-rank test performed to obtain the *p*-value.

### 4.15. Live Cell Imaging Experiments

Live-cell imaging experiments were carried out using a Leica DMI8 inverted microscope with 10× air objective in a chamber at 37 °C, supplied with 5% CO_2_. For *SERPINE1* knock-down in GBM8, time lapse series were captured from randomly selected positions for 5 h of dispersal, with images taken every 5 min. For GBM8 dispersal with tiplaxtinin, time-lapse series were captured from positions for 5 h of dispersal, with images taken in every 60 min. Image stacks were generated for each position and relative increase in dispersal area was measured.

### 4.16. In Vivo Experiments

Non-obese diabetic/severe combined immunodeficiency (NOD/SCID) mice housed and cared in appropriate conditions of the Koç University Animal Facility were used. All protocols were approved by the institution boards of Koç University (ethical code: 2013.198.IRB2.61 and date of permission: 27 August 2013). shControl or sh*SERPINE1* transduced GBM8 cells were further transduced with Firefly Luciferase (Fluc) and mCherry vectors. In addition, 400,000 cells were injected in 7 µL PBS intracranially using stereotaxic injection, as described [60]. Presence and progression of tumors were monitored by repeated noninvasive bioluminescence imaging (IVIS Lumina III, PerkinElmer, Waltham, MA, USA) by injecting D-luciferin (Biotium, Fremont, CA, USA). In addition, 32 days after injection, mice were perfused with 4% PFA, and brains were dissected. Quantification of tumor progression was performed with GraphPad PRISM software (Graphpad Prism v5, San Diego, CA, USA). Furthermore, 10-micron thick cryo-sections from tumors were stained with hematoxylin & eosin and imaged with Leica M205 FA Stereo microscope (Leica Microsystems, Wetzlar, Germany).

## 5. Conclusions

In this study, we provided a motility signature of GBM cells and demonstrated that cell proliferation and migration programs were coupled in dispersal. Despite the accumulating knowledge on biology of invasive cells, there is no directed therapy against these populations in GBMs. To our knowledge, our study provides the first functional demonstration of a direct role of SERPINE1 in GBM cell motility as well as a pro-tumorigenic role in in vivo GBM models. Silencing of *SERPINE1* or its pharmacological inhibition reduced the migration and dispersal of GBM cells in vitro, as well as tumor growth in a primary GBM model in vivo. Therefore, our work identifies SERPINE1 as a potential target for anti-GBM therapies in the future, and inhibition of SERPINE1 may be a promising strategy to attenuate GBM invasiveness.

## Supplementary Materials

The following are available online at https://www.mdpi.com/2072-6694/11/11/1651/s1, Figure S1: U373 cell line-derived spheroids are more dispersive than other GBM cell lines tested, Figure S2: Transcriptome of core and dispersive cells have major differences, Figure S3: *SERPINE1* is upregulated in dispersive cells, Figure S4: Endogenous expression levels of selected EMT genes among cell lines, Figure S5: Effect of *SERPINE1* knock-down with two different shRNAs on dispersal, Figure S6: *SERPINE1* knock-down does not dramatically affect the expression levels of EMT genes, Figure S7: *SERPINE1* inhibitor tiplaxtinin reduces dispersal of additional primary GBM cell lines, Figure S8: Tiplaxtinin does not affect the cell viability of U373 and GBM8 cells, Figure S9: CTGF or CYR61 knock-down reduces the dispersal of U373 spheroids, Figure S10: Knock-down of *CDC45* or *MCM3* reduces U373 cell viability and spheroid dispersal, Figure S11: Vitronectin increases dispersal without changing *SERPINE1* expression, Figure S12: TGFβ is an upstream regulator of U373 dispersal, Figure S13: Original Western blot images and densitometry analysis for Western blot experiments, Table S1: Q-RT PCR primer sequences, Table S2: shRNA sequences, Videos S1–S4: Live-cell imaging experiment videos for control and SERPINE1 knock-down U373 cells with no coating or on vitronectin coating, Videos S5 and S6: Live-cellimagingexperimentvideosforcontrolandSERPINE1knock-downGBM8cells.
