# Supplementary material for: Identification of SERPINE1 as a Regulator of Glioblastoma Cell Dispersal with Transcriptome Profiling
Source: Cancers (Basel). 2019 Oct 25;11(11):1651. doi: 10.3390/cancers11111651 (PMC6896086; doi:10.3390/cancers11111651)
Supplement: Supplementary file 1 [file cancers-11-01651-s001.pdf]

Supplementary Materials

Identification of *SERPINE1* as a Regulator of Glioblastoma Cell Dispersal with Transcriptome Profiling

Fidan Seker, Ahmet Cingoz, İlknur Sur-Erdem, Nazli Erguder, Alp Erkent, Fırat Uyulur, Myvizhi Esai Selvan, Zeynep Hülya Gümüş, Mehmet Gönen, Halil Bayraktar, Hiroaki Wakimoto and Tugba Bagci-Onder

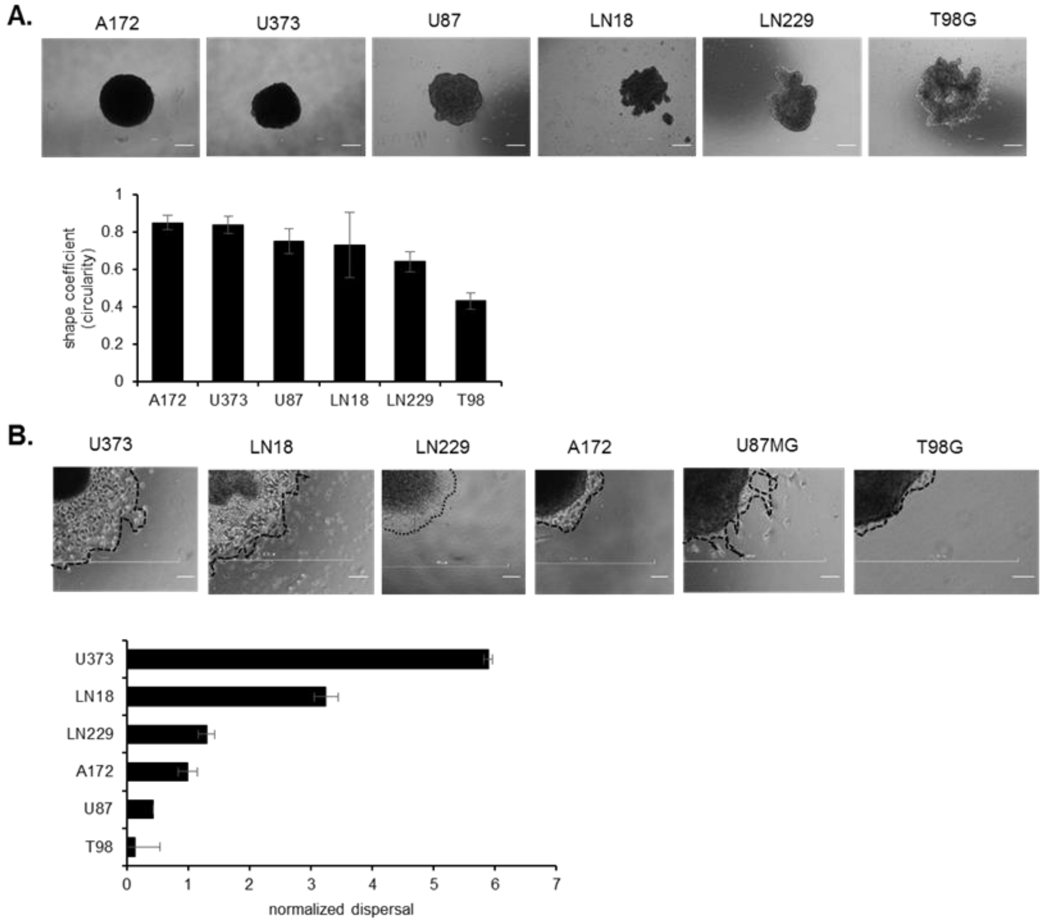

Figure S1. U373 cell line-derived spheroids are more dispersive than other GBM cell lines tested. (**A**) Spheroids of 6 GBM cell lines analyzed for their circularity shape coefficient. Shape coefficient value of 1 indicates perfect spheroids. A172, U373 and U87MG can generate almost perfect spheres (n = 8 spheroids for each cell line, scale bar: 250 μm). (**B**) Dispersal capacity analysis for spheroids at 24 hours of dispersal (n = 8 spheroids for each cell line, scale bar: 250 μm).

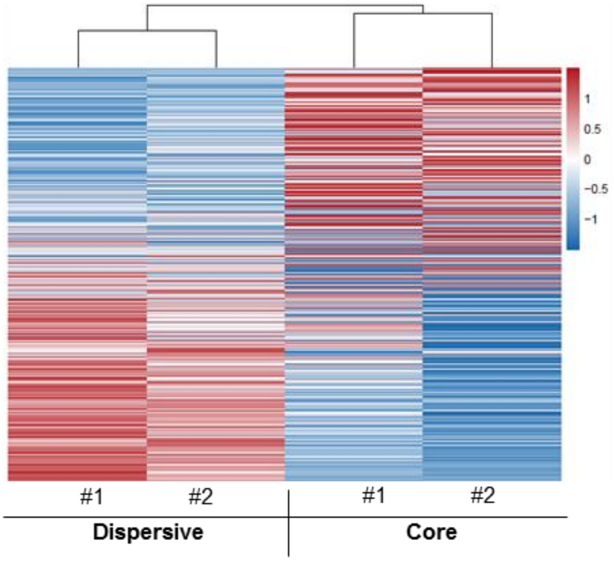

Figure S2. Transcriptome of core and dispersive cells have major differences.

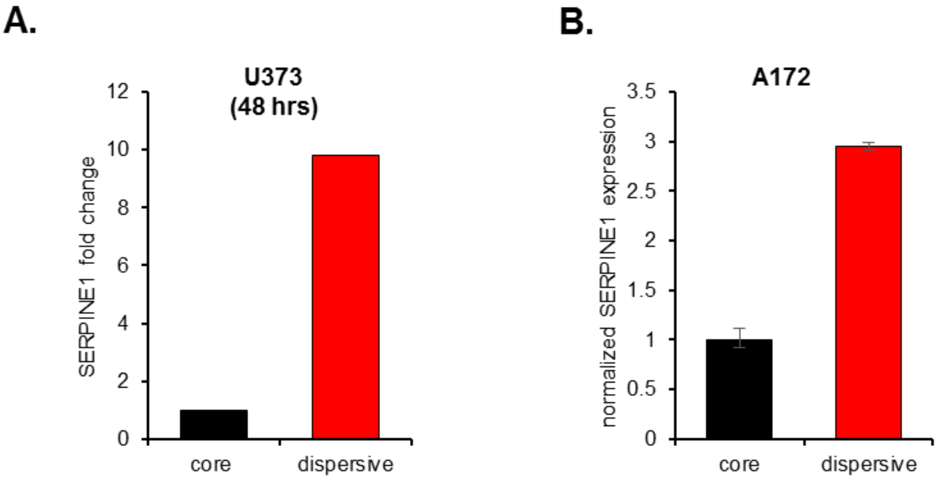

Figure S3. *SERPINE1* is upregulated in dispersive cells. (**A**) *SERPINE1* expression in core and dispersive cells at 48 hours. (**B**) *SERPINE1* expression in core and dispersive A172 cells.

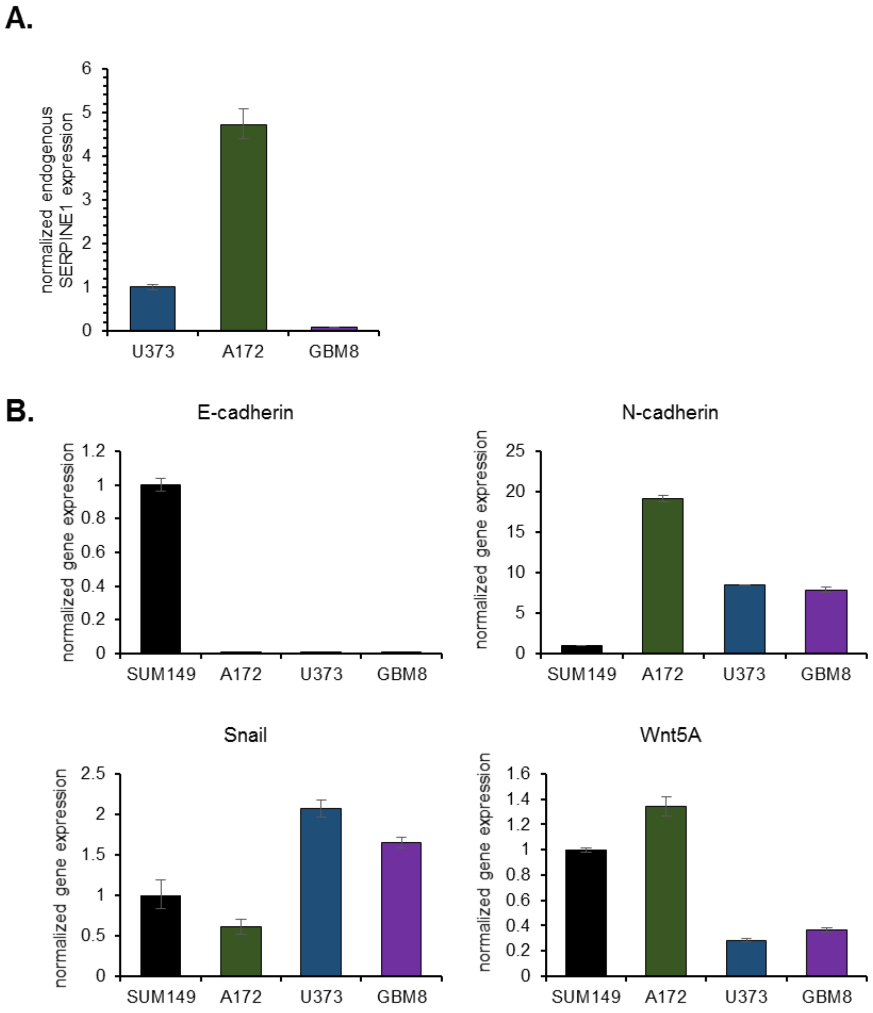

Figure S4. Endogenous expression levels of selected EMT genes among cell lines. (**A**) Endogenous *SERPINE1* expression for U373, A172 and GBM8 cells. (**B**) Endogenous expression of selected EMT genes for epithelial cancer cell line SUM149 and GBM cells A172, U373 and GBM8.

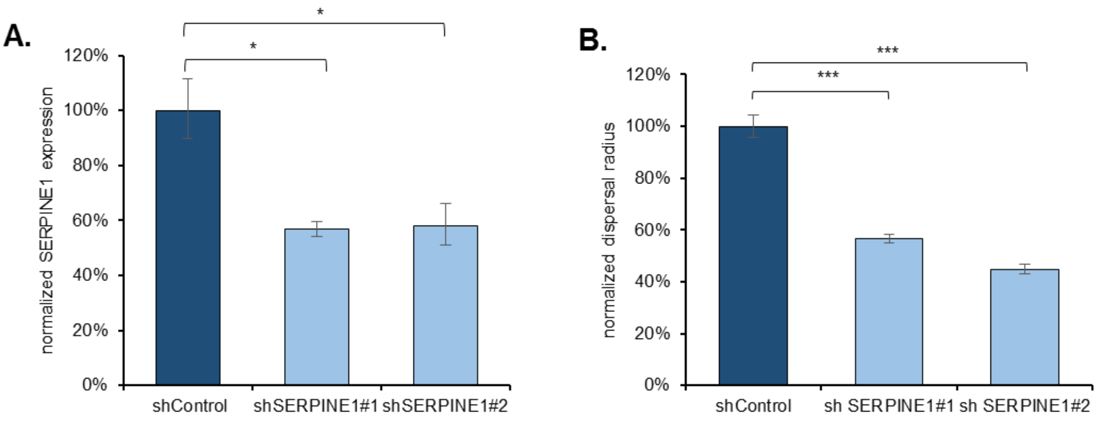

Figure S5. Effect of *SERPINE1* knock-down with two different shRNAs on dispersal. (**A**) mRNA levels of *SERPINE1* with 2 different shRNAs. (**B**) Reduced dispersal with SERPINE1 knock-down (n = 24 spheroids for each condition). (* and *** denote $p < 0.05$ and $p < 0.001$ respectively, two-tailed Student's t-test).

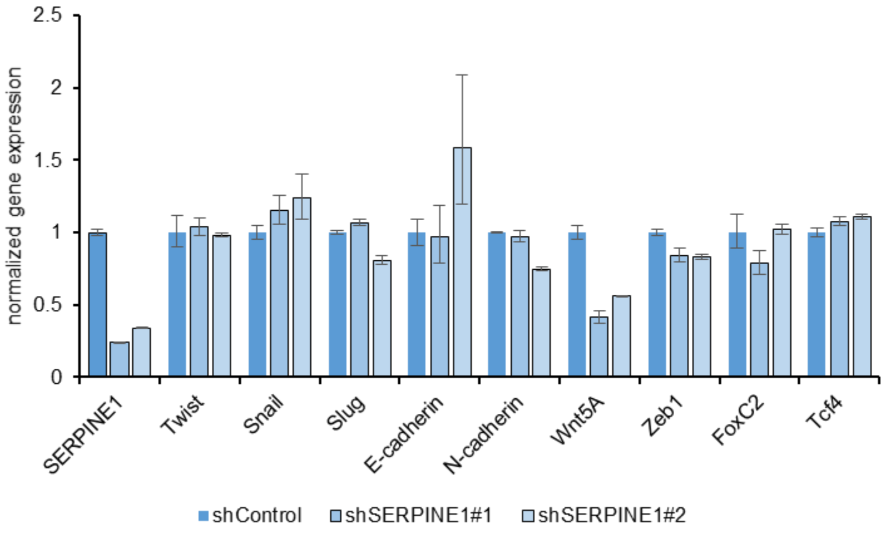

Figure S6. *SERPINE1* knock-down does not dramatically affect the expression levels of EMT genes.

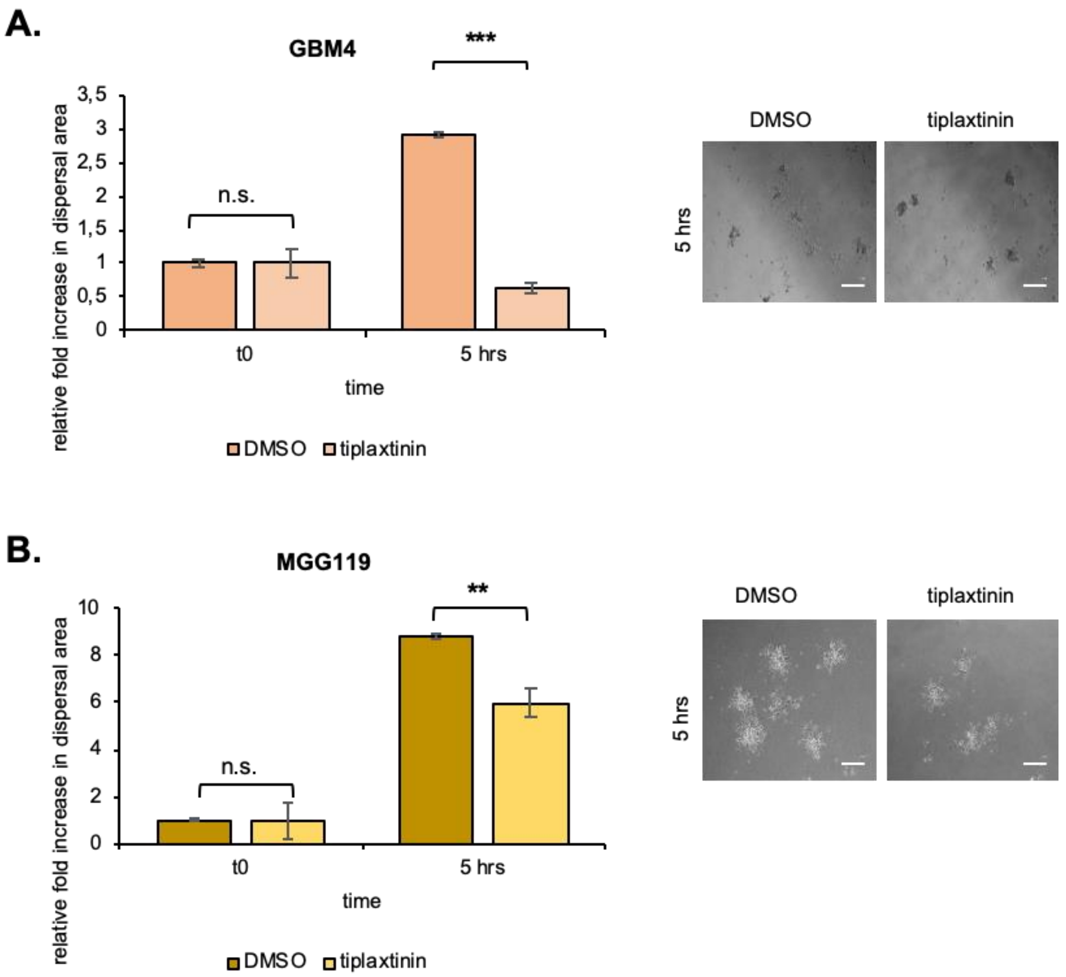

Figure S7. SERPINE1 inhibitor tiplaxtinin reduces dispersal of additional primary GBM cell lines. (**A**) Tiplaxtinin reduces dispersal of GBM4 spheroids (n= 140 spheroids analyzed per condition, scale bar: 140 μm). (**B**) Tiplaxtinin reduces dispersal of MGG119 spheroids (n = 55 spheroids analyzed per condition, scale bar: 140 μm). (** and *** denote $p < 0.01$ and $p < 0.001$ respectively, two-tailed Student's t-test).

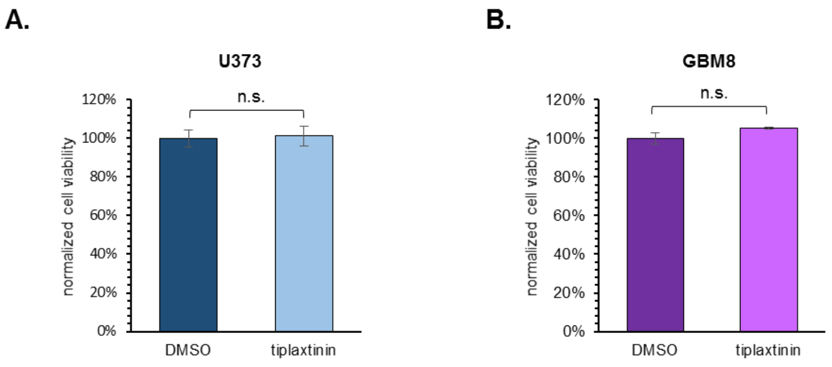

Figure S8. Tiplaxtinin does not affect the cell viability of U373 and GBM8 cells. (**A**) Effect of tiplaxtinin on cell viability for U373 cells (Tiplaxtinin: 300 μM). (**B**) Effect of tiplaxtinin on cell viability for GBM8 cells Tiplaxtinin: 25 μM).

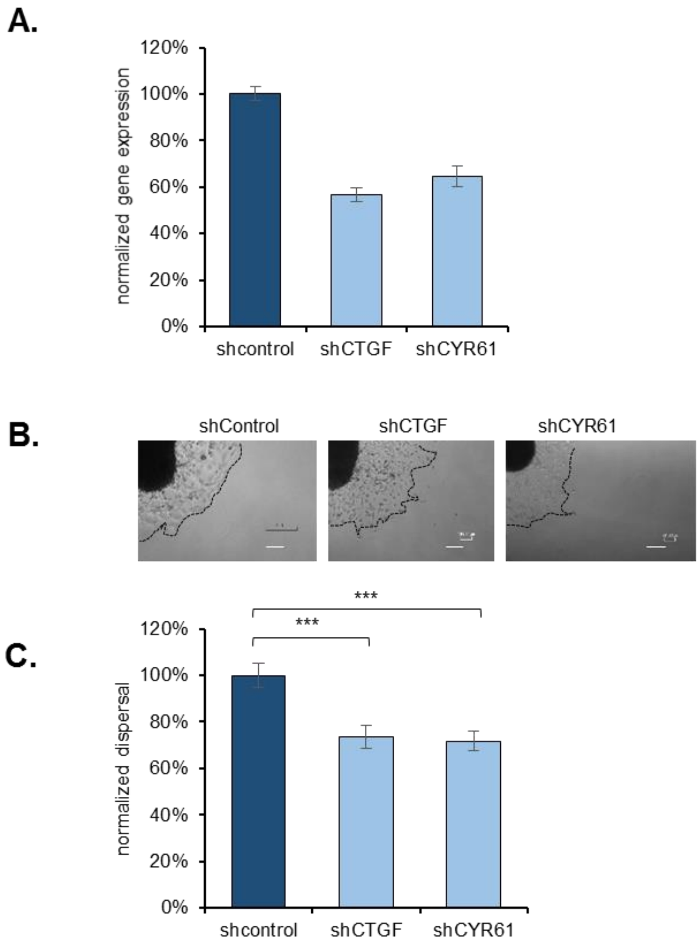

Figure S9. *CTGF* or *CYR61* knock-down reduces the dispersal of U373 spheroids**.** (**A**) mRNA levels after shRNA knock-down of *CTGF* or *CYR61* genes. (**B,C**) Knock-down of *CTGF* or *CYR61* genes reduces dispersal of U373 spheroids significantly (n = 24 spheroids for each condition, scale bar: 200 μm). (*** denotes $p < 0.001$, two-tailed Student's t-test).

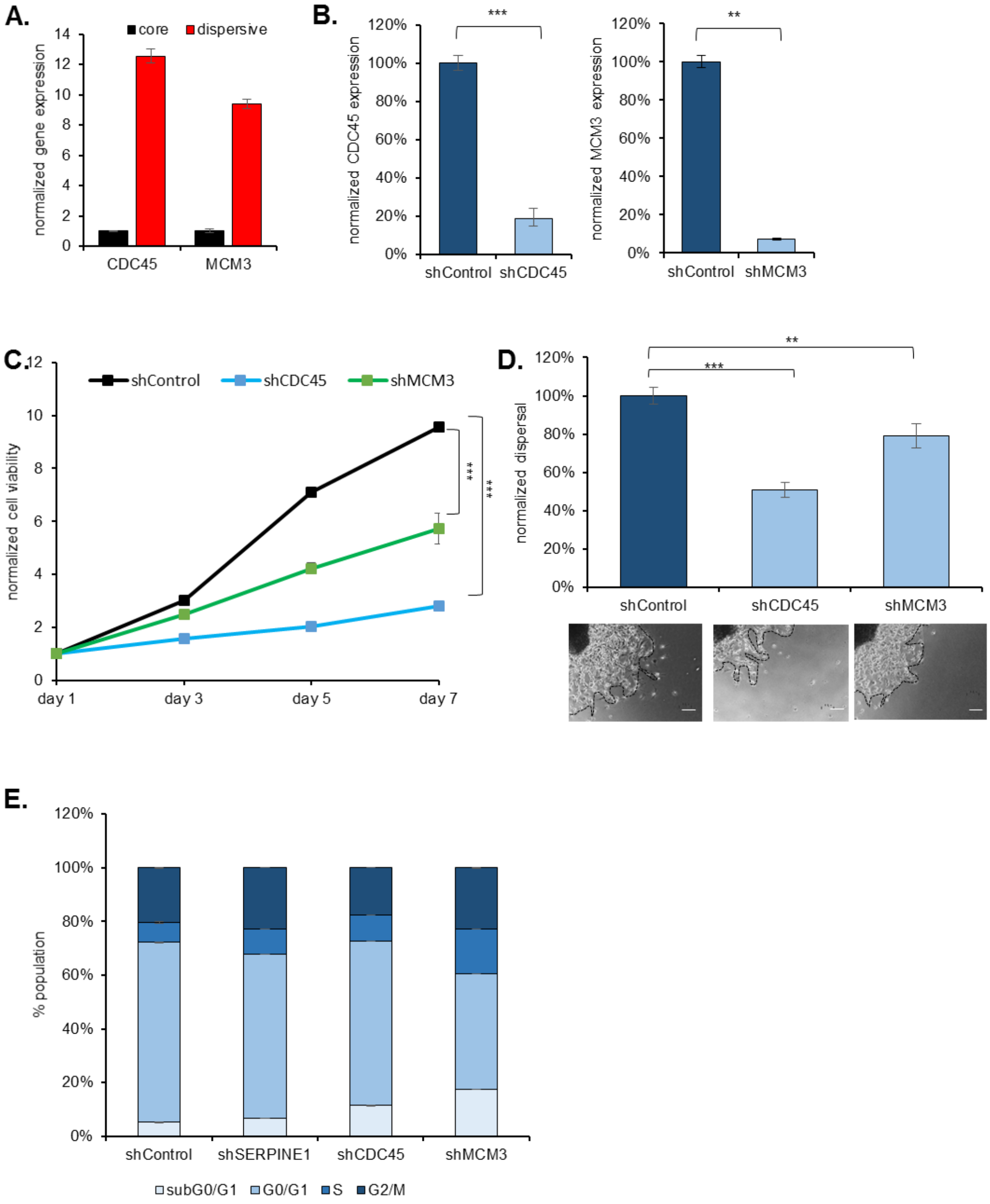

Figure S10. Knock-down of *CDC45* or *MCM3* reduces U373 cell viability and spheroid dispersal (**A**) Expression of *CDC45* and *MCM3* genes in core and dispersive cells. (**B**) mRNA levels after shRNA knock-down. **C.** Knock-down of CDC45 or MCM3 genes reduces viability of U373 cells. (**D**) Knock-down of CDC45 or MCM3 genes reduces dispersal of U373 spheroids significantly (n = 24 spheroids for each condition, scale bar: 200 µm). (**E**) Cell cycle PI flow analysis for CDC45, MCM3 and SERPINE1 knock-down. (** and *** denote $p < 0.01$ and $p < 0.001$ respectively, two-tailed Student's t-test).

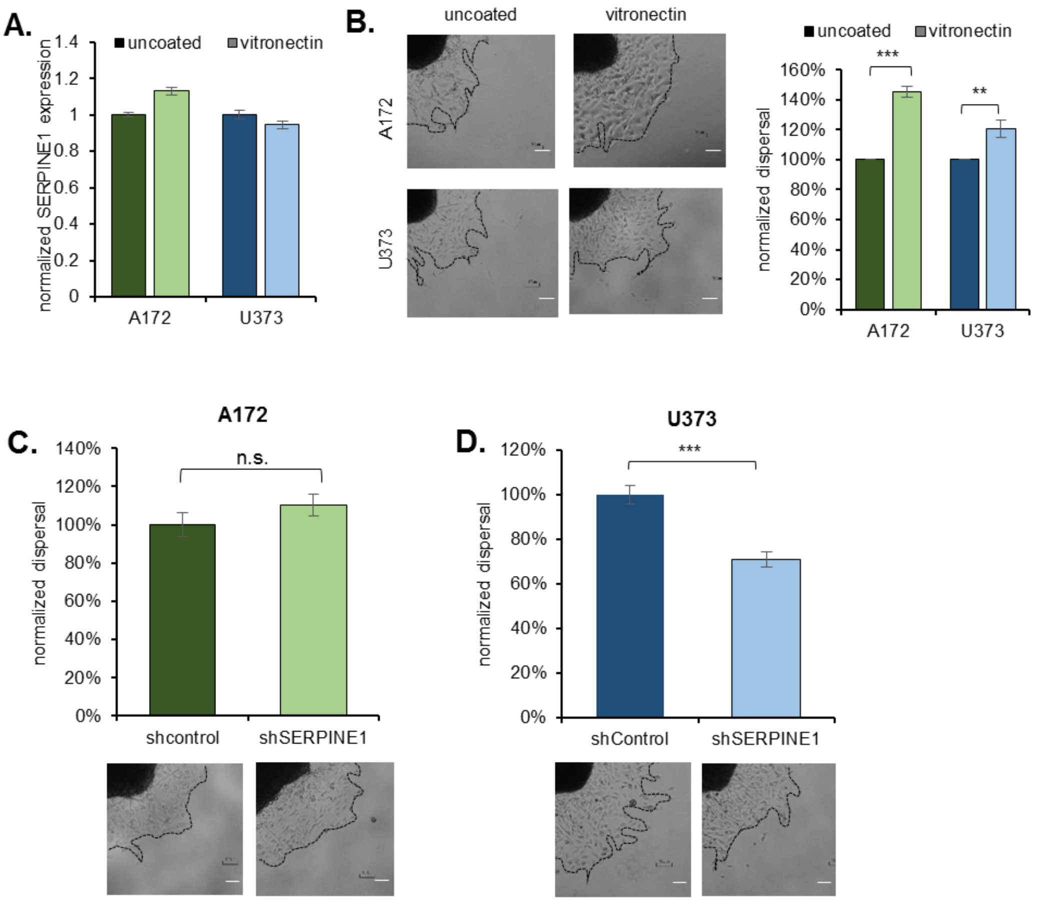

Figure S11. Vitronectin increases dispersal without changing *SERPINE1* expression. (**A**) Comparison of *SERPINE1* expression on no coating and vitronectin coating for A172 and U373 cells. (**B**) Dispersal analysis of A172 and U373 spheroids on no coating and vitronectin coating ($n = 24$ spheroids for each condition, scale bar: 200 μm). (**C**) *SERPINE1* knockdown does not change dispersal of A172 spheroids on vitronectin ($n = 24$ spheroids for each condition, scale bar: 200 um). (**D**) SERPINE1 knockdown reduces dispersal of U373 spheroids also on vitronectin ($n = 24$ spheroids for each condition, scale bar: 200 μm). (** and *** denote $p < 0.01$ and $p < 0.001$ respectively, two-tailed Student's t-test).

A.

Upstream Regulator	Predicted Activation State	Activation z-score
MYC	Activated	7,37
ESR1	Activated	5,59
E2F3	Activated	5,38
MITF	Activated	4,73
E2F1	Activated	4,23
CTNNB1	Activated	4,12
JUN	Activated	3,71
TP63	Activated	3,70
SP1	Activated	3,36
ETS1	Activated	3,11
NOTCH1	Activated	2,97
E2F2	Activated	2,84
SMAD2	Activated	2,71
HOXD3	Activated	2,59
FOSB	Activated	2,41
EGR1	Activated	2,40
HDAC6	Activated	2,38
AR	Activated	2,16
FOSL1	Activated	2,15
SMAD3	Activated	2,05
PPARG	Inhibited	-2,03
NR3C1	Inhibited	-2,32
GLIS2	Inhibited	-2,45
SPDEF	Inhibited	-2,53
HNF4A	Inhibited	-2,70
E2F6	Inhibited	-3,00
HDAC1	Inhibited	-3,10
TP53	Inhibited	-3,12
THRB	Inhibited	-3,16
KLF2	Inhibited	-3,43
RB1	Inhibited	-3,61
NUPR1	Inhibited	-4,75
CDKN2A	Inhibited	-6,26

B.

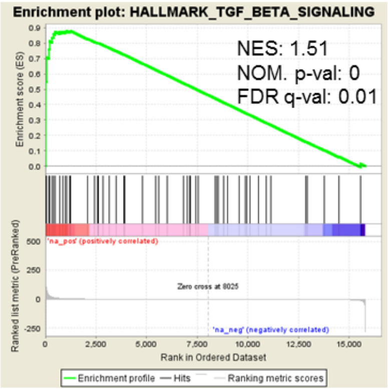

C.

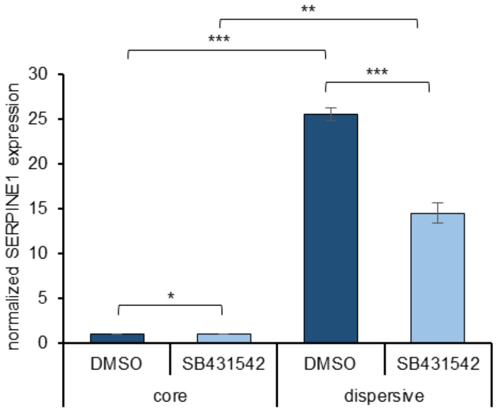

Figure S12. TGFβ is an upstream regulator of U373 dispersal. (**A**) List of upstream regulators activated or inhibited in dispersive cells. (**B**) GSEA enrichment plot for TGFβ signaling in dispersive cells. (**C**) *SERPINE1* induction in dispersive cells reduces in the presence of TGFβ inhibitor SB431542. (*, ** and *** denote $p < 0.05$, $p < 0.01$ and $p < 0.001$ respectively, two-tailed Student's t-test).

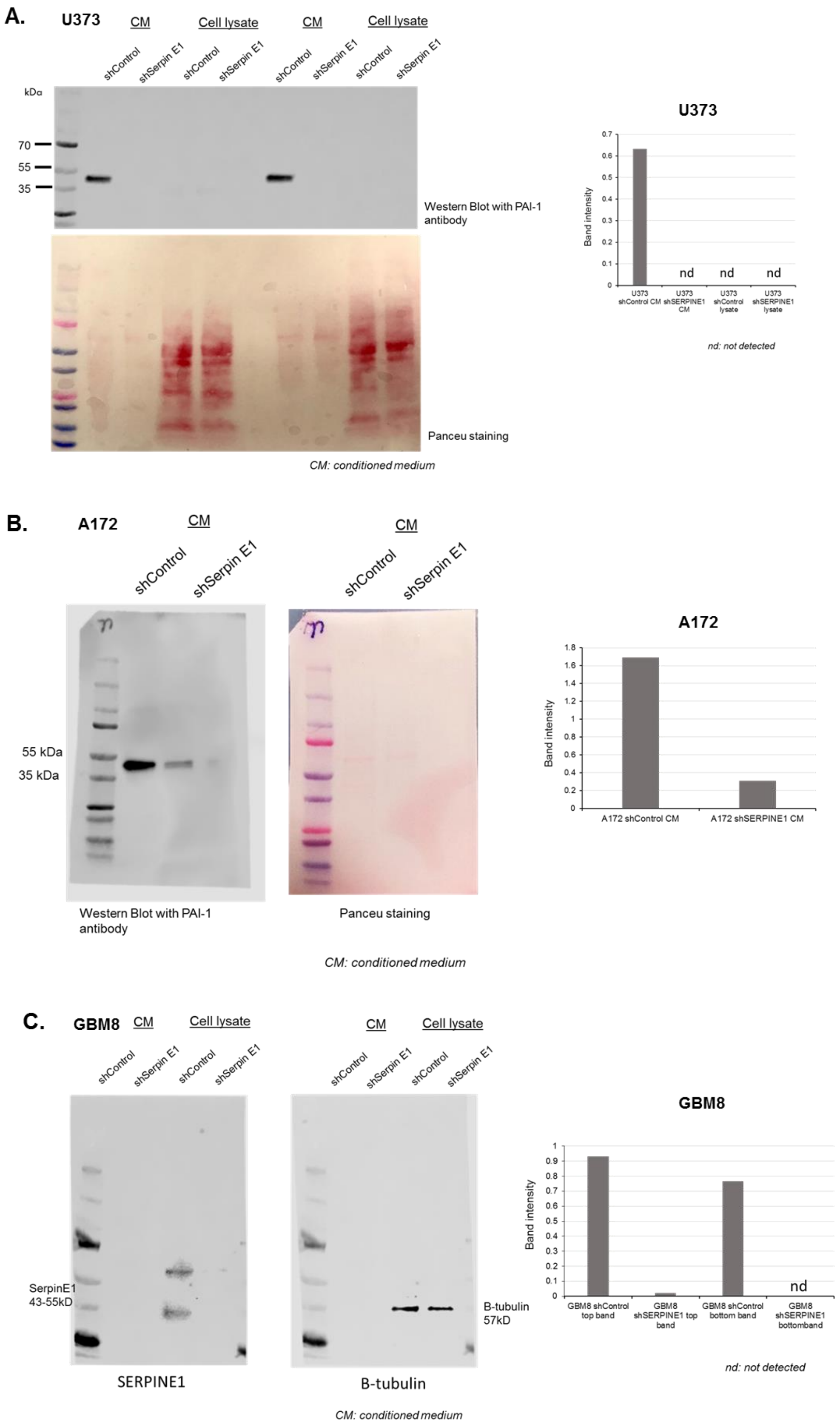

Figure S13. Original western blot images and densitometry analysis for western blot experiments. (**A**) U373 SERPINE1 western blot analysis. (**B**) A172 SERPINE1 western blot analysis. C. GBM8 SERPINE1 western blot analysis.

Table S1. Q-RT primer sequences.

Gene Name	Forward Primer (5′-3′)	Reverse Primer (5′-3′)
GAPDH	AGCCACATCGCTCAGACAC	GCCCAATACGACCAAATCC
SERPINE1	TCGAGGTGAACGAGAGTGGCA	AAGGACTGTTCCTGTGGGGTTGT
CYR61	CCAAGAAATCCCCCGAACCA	GAAACGCTGCTTCATTGGCA
CCND1	AAGATCGTCGCCACCTGGAT	AGCTCCATTTGCAGCAGCTC
CTGF	CACCCGGGTTACCAATGACA	GGATGCACTTTTTGCCCTTCTTA
CSF2	GCTGAGATGAATGAAACAGTAGAAG	CTGGGTTGCACAGGAAGTT
INHBA	GAAGAGTGGGGACCAGAAAGAGAAT	GCAGCGTCTTCCTGGCTGTT
CXCL8	TTGGCAGCCTTCCTGATTTCT	ATTCTCAGCCCTCTTCAAAAACTTC
ANKRD1	ACGCCAAAGACAGAGAAGGAGAT	AGATCCATCGGCGTCTTCCCA
NAV3	CATCCTCCCAAAGATCTTCGCATCA	TCAGCTCACTTCCTCTAGAGTTCAC
RAD51	TGCGGACCGAGTAATGGCA	TCCTTCTTTGGCGCATAGGCA
HAP1	AGCTGGCTTCGGAGAAGGAAA	AAATCATACCTTAGGCTGGATGTGT
EFNA1	AGTTCCAGCGCTTCACACCTT	TGGGTCATCTGCTGCAAGTCTCT
YPEL4	GGAGCAGACCTCAAGGTGACTT	TGAAGCAGCGGAGCAGGTTG
BMF	GAGCCATCTCAGTGTGTGGAG	GCCAGCATTGCCATAAAAGAGTC
RGS16	TCAGAGCTGGGCTGCGATACT	TTCAGGAAAGCGTGGAAGGCA
PTP4A3	CCGGTGGAGGTGAGCTACAA	GCCAGTCCACAACGGTGAT
PCK1	GACACAGTGCCCATCCCCAAA	CGTCAGCTCGATGCCGATCTT
PTX3	CAGACGCGAGCCGACCTG	TGGTCTCACTGGATGCACGCT
NTM	TGGTACAAGGATGACAAAAGACTGA	GGGGTCAGGGCTGTAGTTTTCA
CDC45	TGACAGTGATGGGTCAGAGCCT	GTTCACTCCCAGAGCCACTCC
MCM3	AGGTAGTTCTTTGGCAGCGG	AAATTCCCTGGTCTTCCTCGT

Table S2. shRNA sequences.

shRNA	Oligo Sequence (5′-3′)
shSERPINE1 #1	TGCTGTTGACAGTGAGCGAGGACACCCTCAGCATGTTCATTAGTGAAGCCACA GATGTAATGAACATGCTGAGGGTGTCCCTGCCTACTGCCTCGGA
shSERPINE1 #2	TGCTGTTGACAGTGAGCGCCCATACAATTTCATCCTCCTTTAGTGAAGCCACA GATGTAAAGGAGGATGAAATTGTATGGTTGCCTACTGCCTCGGA
shCDC45	TGCTGTTGACAGTGAGCGACCAGTCAATGTCGTCAATGTATAGTGAAGCCACA GATGTATACATTGACGACATTGACTGGCTGCCTACTGCCTCGGA
shMCM3	TGCTGTTGACAGTGAGCGACCACAGATGATCCCAACTTTATAGTGAAGCCACA GATGTATAAAGTTGGGATCATCTGTGGCTGCCTACTGCCTCGGA
shCTGF	TGCTGTTGACAGTGAGCGCCGCCTCCTGCAGGCTAGAGAATAGTGAAGCCAC AGATGTATTCTCTAGCCTGCAGGAGGCGTTGCCTACTGCCTCGGA
shCYR61	TGCTGTTGACAGTGAGCGACCTGTGAATATAACTCCAGAATAGTGAAGCCACA GATGTATTCTGGAGTTATATTCACAGGGTGCCTACTGCCTCGGA

Videos S1–S4: Live-cell imaging experiment videos for control and *SERPINE1* knock-down U373 cells with no coating or on vitronectin coating. Experiment was carried out using Leica DMI8 inverted microscope with 10× air objective in a chamber at 37°C, supplied with 5% CO_2. Time lapse series were captured from positions for 21 hours 55 mins, images were taken in every 11 minutes.

Videos S5–S6: Live-cell imaging experiment videos for control and *SERPINE1* knock-down GBM8 cells. Experiment was carried out using Leica DMI8 inverted microscope with 10X air objective in a chamber at 37 °C, supplied with 5% CO_2. Time lapse series were captured from positions for 5 hours of dispersal, images were taken in every 5 minutes.

© 2019 by the authors. Licensee MDPI, Basel, Switzerland. This article is an open access article distributed under the terms and conditions of the Creative Commons Attribution (CC BY) license (http://creativecommons.org/licenses/by/4.0/).
